# Impact of Ramadan Intermittent Fasting on the Heart Rate Variability and Cardiovascular Parameters of Patients with Controlled Hypertension

**DOI:** 10.1155/2021/6610455

**Published:** 2021-03-29

**Authors:** Sabah Hammoud, Iman Saad, Rita Karam, Fayez Abou Jaoude, Bart J. F. van den Bemt, Mazen Kurdi

**Affiliations:** ^1^Laboratory of Experimental and Clinical Pharmacology, Faculty of Sciences, Section 1, Lebanese University, Rafic Hariri Educational Campus, Hadat, Lebanon; ^2^Department of Pharmacy, Radboud University Medical Center, Nijmegen, Netherlands; ^3^Department of Cardiology, Rafic Hariri University Hospital, Beirut, Lebanon; ^4^Department of Pharmacy, Sint Maartenskliniek, Nijmegen, Netherlands; ^5^Department of Pharmacy, University Medical Center Maastricht, Maastricht, Netherlands

## Abstract

**Background:**

Conflicting results are reported on the effect of Ramadan fasting on the cardiovascular health of patients with hypertension, a highly prevalent cardiovascular disease risk factor. This research aimed to evaluate the impact of fasting on cardiac health and heart rate variability (as a measure of cardiac stress) of hypertensive patients.

**Methods:**

Patients with controlled hypertension were followed in a prospective cohort during and after Ramadan. Lipid panel and blood glucose were measured at the end of each phase. Blood pressure and heart rate variability were monitored in the morning, afternoon, and evening of each follow-up day.

**Results:**

The study included 58 subjects (mean age: 54 ± 11.5 years, 52% male). Fasting did not affect body composition, lipid panel parameters, and blood pressure of hypertensive subjects; males only presented lower body weight and hip circumference during Ramadan. Blood glucose was significantly higher during Ramadan. Fasting significantly increased HRV during the afternoon period.

**Conclusions:**

Ramadan intermittent fasting reduces cardiac stress among hypertensive patients controlled by and adherent to hypertensive medication, without affecting their hypertensive state.

## 1. Introduction

Ramadan constitutes the annual fasting period for over a billion Muslims worldwide, during which people totally abstain from food and drink from sunrise until sunset. During Ramadan, the lifestyle of these fasting people changes temporarily, affecting their eating pattern, sleeping periods, physical activity, and smoking habits [1]; these alterations may in turn affect the health status of patients suffering from cardiovascular diseases and its risk factors (hypertension, diabetes, dyslipidemia, and imbalanced autonomic nervous activity) [2].

One of the highly prevalent cardiovascular disease risk factors is hypertension, which occurs between 18% and 27% worldwide depending on the region [3]. Hypertensive heart disease is considered a leading risk for early deaths and disability [4] and, according to the global health estimates, is ranked among the top 20 causes of mortality worldwide, accounting for 1.6% of total deaths in 2016 [5].

Limited number of studies (5 research manuscripts as for April 2020) demonstrated the effect of Ramadan fasting on blood pressure, and cardiovascular parameters in hypertensive patients, who adhered to their antihypertensive medications [6–10]. Previous research was diverse in terms of hypertension grade of subjects that ranged from pre-hypertensive state [11] to grade 3 hypertension [7]. In addition, different inclusion criteria were applied (such as diabetes, cardiac hypertrophy, sinus arrhythmia, renal disease, etc.), and different fasting durations were studied (from 12 hours [7] to 16 hours [10]). Of note, some cardiac outcomes (such as lipid panel [8] and autonomic nervous system [10]) were only evaluated once with a relatively low sample size.

Previous review studies concur that Ramadan fasting has no significant effect on blood pressure measurements of hypertensive subjects [12, 13] and claim that fasting may be a safe practice for patients with controlled hypertension [2]. On the other hand, conflicting results were reported for the variation in body weight [6, 7, 9], as well as in heart rate [9, 10], of hypertensive patients during Ramadan fasting.

Additionally, few studies described an improvement in lipid panel, as evidenced by a significant increase in high density lipoproteins (HDL) and decrease in triglycerides and low density lipoproteins (LDL); also an enhancement in oxidative stress profile was observed, reported as an increase in the antioxidant glutathione and a decrease in the reactive compound malondialdehyde, an oxidative stress biomarker [8].

In a recent study, Mzoughi et al. also assessed the autonomic nervous activity during Ramadan and claimed decreased heart rate variability and enhanced sympathetic activity among hypertensive patients with sinus rhythm, who varied significantly in terms of lifestyle characteristics [10].

To our knowledge, this is the first study to collectively address multiple cardiac health parameters among the same group of hypertensive subjects with homogenous characteristics during Ramadan fasting. This research aims to evaluate the intraday and interday effect of fasting on cardiac health at the level of the autonomic nervous system activity (heart rate variability), lipid profile, blood pressure, and body composition among patients with controlled hypertension.

## 2. Materials and Methods

### 2.1. Study Design

In this observational prospective cohort, participants were followed for 24 h under two conditions: fasting state during the last two weeks of Ramadan, and nonfasting state one month after Ramadan. Participants were instructed to follow their regular medication posology and their routine activities in both monitoring phases.

### 2.2. Study Participants

The study was done according to the Declaration of Helsinki, with an IRB approval granted from Rafic Hariri University Hospital (Beirut, Lebanon). All participants signed an informed consent form prior to their enrollment. Participants were considered eligible if over 18 years old, with controlled hypertension by the use of antihypertensive medications (SBP ≤ 129 mmHg and DBP < 80 mmHg) [14], and willing to voluntarily fast for at least 15 days of Ramadan. Patients who had chronic CVD, patients who underwent surgeries in the past two years, and pregnant women were excluded from the study.

### 2.3. Data Collection

Data was collected by means of individualized visits, preserving the natural setting for participants in which they followed their routine activities.

#### 2.3.1. Anthropometric Measurements

Morning baseline measurements were recorded for the body composition of participants at each visit. The body height and waist and hip circumferences (cm) were measured by a regular meter. BF511 body composition monitor (Omron Healthcare, Lebanon) was used to evaluate the body weight (kg), body mass index (BMI) (kg/m^2^), total body fat (%), visceral fat level, skeletal muscle (%), and resting metabolism (kcal).

#### 2.3.2. Blood Pressure Measurement

Blood pressure was measured using an Omron M3 HEM-7131-E blood pressure monitor (Omron Healthcare, Lebanon). Measurements were recorded at four time points (during/after Ramadan) after a 10–15 min rest in the sitting posture: (1) baseline morning measurement, (2) one hour before breaking fast/before having lunch, (3) one hour after breaking fast/after having lunch, and (4) terminal morning measurement.

#### 2.3.3. Blood Test Analysis

Blood samples for all participants were collected at the end of each phase and analyzed for lipid profile (triglyceride, total cholesterol, LDL, HDL, non-HDL (mg/dl)) and blood glucose levels (mg/dl).

#### 2.3.4. Cardiac Monitoring and Heart Rate Variability Analysis

Participants were monitored for ECG over 24 h in fasting and nonfasting conditions using validated and medical CE certified Bittium Faros cardiac monitoring device 360° (Bittium Biosignals, Kuopio, Finland) at a sampling rate of 250 Hz; monitoring was done through 1-channel, 3-electrode cable set, attached to the skin by Ambu® WhiteSensor WS adhesive electrodes (Ambu®, Copenhagen, Denmark). Heart rate variability (HRV) was then analyzed using HRV Scanner Professional software V. 3.05.13 (Bittium Biosignals, Kuopio, Finland). Short-term HRV was analyzed over 5 min interval at 3 times points matching blood pressure measurements while participant is at rest (i.e., (1) one hour before breaking fast/before having lunch, (2) one hour after breaking fast/after having lunch, and (3) terminal morning measurement).

#### 2.3.5. Questionnaire

A structured questionnaire was filled during and after Ramadan regarding the lifestyle and monitoring days' activities of participants. The questionnaire was designed and piloted by the researchers prior to implementation of the study, after which a final version was developed. It covered baseline demographics, medical history, dosing regimens of administered medications during and after Ramadan, smoking status, physical activities, meal consumption, and sleeping pattern.

### 2.4. Statistical Analysis

Statistical analysis was done using IBM SPSS statistics V.20.00. Results are presented as mean ± standard error mean (SEM), and statistical significance was considered at *p* value <0.05. Paired *t*-test was used to compare mean difference between during and after Ramadan. Repeated measures of ANOVA was done to compare within monitoring day variations.

## 3. Results

### 3.1. Population Determinants

The study included 58 participants (30 male, 28 female) with a mean age of 54.3 ± 11.5 years. Baseline characteristics of participants are illustrated in Table 1; the majority were married (84.5%) and employed (62.1%). Almost all participants consumed caffeine on a daily basis, with 44.9% having 3 or more cups of caffeinated drinks per day. Most of the participants presented sedentary lifestyle (79.3%) and were almost equally distributed between smokers and nonsmokers.

Patients varied in terms of hypertension condition duration, with 63.8% being diagnosed with hypertension for more than 5 years, and a minority (6.9%) diagnosed during the last year. More than half of the participants reported having dyslipidemia (65.5%), and almost a quarter reported having diabetes (24.1%). Patients followed either a mono-therapy for hypertension (32.8%), or combinational therapy of 2 or 3 drugs (63.8%). Therapies included different classes of antihypertensive drugs, with beta blocker and angiotensin II receptor blockers being the most utilized (44.8% and 41.4%, respectively).

### 3.2. Effect of Ramadan Fasting on Body Composition

Body composition parameters were clustered per gender (Table 2). Females did not present any significant change in body composition in response to fasting. Males presented significantly lower body weight (mean difference 1.07 kg, *p* value = 0.045) and BMI (mean difference 0.37 kg/m^2^, *p* value = 0.037) during Ramadan, as well as lower hip circumference (*p* value = 0.023) without affecting waist-hip ratio. Both genders were obese and carried an unfavorable body composition profile, presenting very high BMI (>30 kg/m^2^), waist-hip ratio (male >0.9, female >0.85), and total body fat (male >28%, female >40%), and low skeletal muscle levels (male <33.1%, female <24.1%). Females presented high visceral fat levels (10–14), while males presented very high levels (15–30).

### 3.3. Effect of Ramadan Fasting on Lipid Profile and Blood Glucose Levels

Lipid profile and blood glucose levels are reported in Table 3. Mean triglyceride levels were elevated (>160 mg/dl presented by 48.27% of patients during and after Ramadan), and mean HDL cholesterol levels were normal within the lower borderline, yet yielding a high total cholesterol to HDL cholesterol ratio (>5 in 37.93% and 36.21% of subjects during and after Ramadan, respectively). Fasting did not significantly alter any of the lipid panel parameters (*p* value >0.05). Blood glucose level was significantly higher in fasting state (mean difference 6.65 mg/dl, *p* value = 0.023), lying in upper borderline levels in both conditions.

### 3.4. Effect of Ramadan Fasting on Blood Pressure Variation

Subjects presented normotensive profile in fasting and nonfasting conditions. SBP and DBP were comparable throughout a fasting and nonfasting day; also, no significant difference was observed between fasting and nonfasting condition per measurement time point (Table 4, *p* value >0.05).

### 3.5. Effect of Ramadan Fasting on Heart Rate Variability

**Figure 1:**
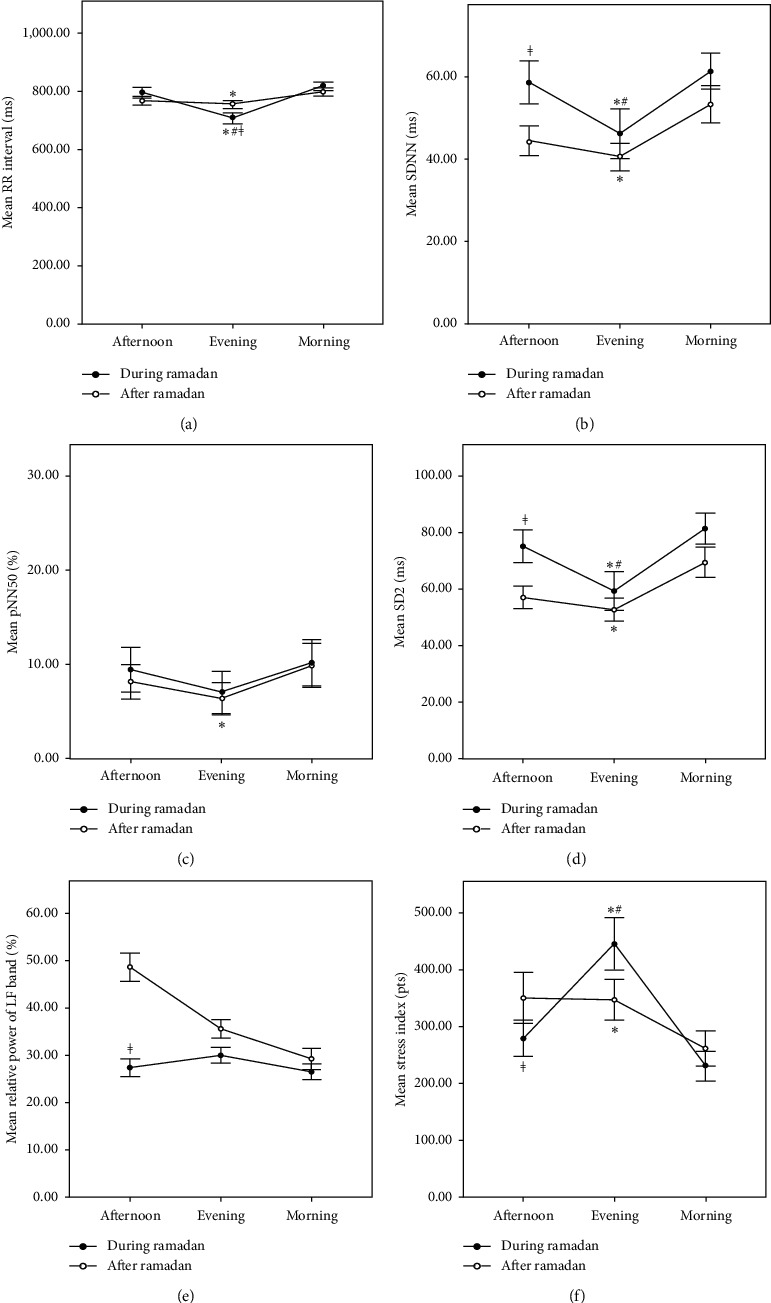
Variation of heart rate variability parameters significantly affected by Ramadan fasting. Graph showing variation of heart rate variability parameters during and after Ramadan, which have been significantly affected in fasting condition: (a) R-R interval (ms), (b) SDNN (ms), (c) pNN50 (%), (d) SD2 (ms), (e) relative power of LF band (%), and (f) stress index (pts). ^*∗*^Statistically different compared to morning period of the same day, ^#^statistically different to afternoon period of the same day, ^≠^significantly different compared to same period of non-fasting day.

Heart rate variability parameters measured at three time points per monitoring day are reported in Table 5. During Ramadan, morning and afternoon periods were comparable and showed significantly higher RR interval, SDNN, and SD2, and lower stress index versus the evening period of the same day (Figures 1(a), 1(b), 1(d), and 1(f); *p* value <0.05). However, after Ramadan, only morning period was significantly different from the evening period, with morning presenting higher RR interval, SDNN, pNN50, and SD2, and lower stress index (Figures 1(a)–1(d); *p* value <0.05); afternoon period was not statistically different from both morning and evening phases (*p* value >0.05).

Upon comparing the same time point in two different conditions, HRV during morning period was similar in fasting and nonfasting condition. In the afternoon, fasting significantly reduced HRV and presented significantly higher SDNN, SD2, and SD2/SD1 ratio, and lower stress index and LF component (Figures 1(b), 1(d), 1(e), and 1(f); *p* value <0.05), suggesting lower sympathetic input. In the evening, subjects only presented significantly lower RR interval and SD2/SD1 ratio in fasting state (*p* value <0.05), implying a possible decrease in sympathetic activity without affecting HRV.

## 4. Discussion

This research illustrates the effect of Ramadan fasting on the cardiac health of patients with hypertension, treated with different combinations of antihypertensive medications. Fasting did not affect body composition, lipid panel parameters, or blood pressure of hypertensive subjects; males only presented lower body weight and hip circumference during Ramadan. Blood glucose was significantly higher during Ramadan. Of note, fasting significantly increased HRV during the afternoon period.

The patients presented several CVD risk factors, including obesity and unfavorable body composition as shown by high levels of total and visceral fat versus the low percentage of skeletal muscle, and accompanied with very high BMI. Despite this unfavorable profile, body composition did not show any significant change in response to fasting, even though fasting significantly reduced BMI of males exclusively. Although fasting was shown to reduce the body weight and ameliorate the body composition of normotensive healthy adults [15, 16], results of previous studies regarding hypertensive subjects remain controversial. Two studies reported no significant change in body weight of hypertensive subjects [7, 9], which is consistent with our results for the whole group (*p* value >0.05, data not shown), and females alone. Perk et al. reported a significant reduction in body weight during fasting, observed among 17 patients out of which 88% are male [6], which seems consistent with our male results. Norouzy et al. also reported a loss of fat mass in healthy individuals, which was significant in males only [1]. This supports the necessity of further research on body composition, and the interaction effect of gender.

Dyslipidemia and high blood glucose levels are also considerable CVD risk factors. Our results revealed no significant effect on any lipid profile parameters, similar to that observed during and after Ramadan among patients with moderate hypertension on 2 or less antihypertensive drugs, and having a similar lipid profile to our results [8]. However, Al-Shafei reported that fasting induced a drop in LDL and an increase in HDL when compared to before the Ramadan period, which was maintained 6 weeks post-Ramadan, unlike the reversible drop in triglycerides [8]. The enhancement in lipid panel parameters as a function of fasting, observed by Al-Shafei, agrees with the favorable results reported among healthy population [17, 18]. Despite the fact that only 24% of patients reported having diabetes, fasting blood glucose appeared within the upper bound limits, which were significantly higher during Ramadan; this may be explained by the carbohydrate-rich meals consumed during Ramadan, and shorter overnight fasting hours prior to blood sample collection due to the sohour meal, a light meal consumed before sunrise during Ramadan. Further studies should investigate fasting effect on the biochemical profile to better understand the confounding factors affecting its variations.

Patients adhered to their antihypertensive medication posology (dose and frequency) during Ramadan and presented well-controlled blood pressure levels, lying within the normal recommended values [14], which tended to decrease between the morning and evening phases, and were comparable to nonfasting day. These results are consistent with previous findings that found no significant change in blood pressure levels during and after Ramadan [7, 9], and comparable to those reported before and during Ramadan [6, 9]. This is opposite to the fasting-induced decrease in blood pressure levels observed among patients with SBP 150–180 mmHg and/or DBP 95–120 mmHg [8]; despite the reduction reported by Al-Shafei [8], SBP and DBP did not reach normal recommended levels and were not controlled by use of 2 or less antihypertensive medications.

To our knowledge, this is the first study to report intraday variation of HRV among hypertensive patients in fasting state. Only one previous study addressed HRV among hypertensive subjects during Ramadan, but was limited to interday variation during and after Ramadan [10]. Mzoughi et al. observed a decrease in HRV, as evidenced by lower SDNN and SDANN (representative of whole HRV), and a further enhancement in the sympathetic nervous system upon fasting among hypertensive subjects suffering from sinus rhythm when compared to after the Ramadan period [10]. Mzoughi et al. study was done on a relatively low number of subjects (*n* = 20), who presented diverse characteristics in terms of smoking habits, antihypertensive medication classes, and combination therapies, and 20% presenting cardiac hypertrophy, which could be confounding factors. On the contrary, our results revealed an increase in HRV, and thereby a lower cardiac stress, only during the afternoon period during Ramadan, i.e., after prolonged fasting hours, compared to nonfasting day after Ramadan. Our results are similar to those reported among healthy individuals with sinus rhythm, who presented higher HRV during Ramadan compared to the first week after Ramadan ended [19]. HRV in the morning and evening periods were comparable during and after Ramadan, with the evening period during Ramadan showing only higher heart rate and SD2/SD1 ratio, which may be suggestive of slight increase in sympathetic nervous activity without affecting the overall HRV changes.

On the other hand, intraday analysis revealed that evening period showed lower HRV as evidenced by lower SDNN (sympathetic nervous system indicator) and SD2 (long term HRV marker) and higher stress levels compared to morning and afternoon period during Ramadan, and only to morning period after Ramadan. Our results during Ramadan are comparable to the circadian HRV changes observed among healthy female adults, who fasted for similar duration (16 hours) [20]. This may imply that, in nonfasting condition, there is a tendency to gradual decrease in HRV from the morning till the evening time as a result of food ingestion all day and metabolic activity needs, while upon fasting during Ramadan, the decrease in HRV is delayed until the evening period upon breaking fast and initiating the metabolic activities of the body after long hours of food deprivation. Further research should investigate the autonomic nervous activity of hypertensive subjects in response to Ramadan fasting to better describe the induced changes and elaborate the underlying mechanism behind changes in sympathetic and parasympathetic activities.

Overall, this study presents a novel finding regarding the intraday variations of the autonomic nervous activity as function of fasting. However, it lacks measurement before Ramadan, which may have been beneficial for analyzing fasting effect on lipid profile and blood glucose variations. Also, subjects were diverse in terms of antihypertensive medication combinations, which restricted a possible stratification of patients according to drug classes, knowing that beta blockers and calcium channel blockers have negative inotropic effects.

## 5. Conclusions

In conclusion, the present study provides evidence for lower cardiac stress upon prolonged hours of intermittent fasting, with controlled blood pressure levels during Ramadan fasting. This implies that Ramadan fasting may be a risk-free practice for patients adhering to their antihypertensive medication. Nonetheless, hypertensive patients are prone to developing further CVD by presenting a dyslipidemia profile, high blood glucose levels, and an unfavorable body composition, which were unaltered upon fasting, yet need further management to avoid undesired sudden cardiac-related events in fasting and nonfasting conditions.

This work may be complemented by including a larger sample size that allows for stratification of the results based on antihypertensive medication classes, and to study the interaction of possible confounding factors such as smoking and physical activity.

## Figures and Tables

**Table 1:** Characteristics of included participants in terms of demographics, lifestyle, and disease history.

	Parameter	Frequency (percentage) (total participants, *N* = 58)
Demographics	*Gender*	
	Male	30 (51.7)
	Female	28 (48.3)
	Age (years ± SD)	54.27 ± 11.48
	*Marital status*	
	Single	2 (3.4)
	Married	49 (84.5)
	Widowed	7 (12.1)
	*Highest obtained degree*	
	None	1 (1.7)
	Middle school	20 (34.5)
	High school	14 (24.1)
	University	23 (39.7)
	*Occupational status*	
	Unemployed	21 (36.2)
	Employed	36 (62.1)
	Retired	1 (1.7)

Lifestyle	*Cups of caffeinated drinks per day*	
	None	1 (1.7)
	1-2 cups	26 (44.8)
	3-4 cups	19 (32.8)
	≥5 cups	7 (12.1)
	Rarely	5 (8.6)
	*Physical activity*	
	None	46 (79.3)
	Jogging	5 (8.6)
	Weight lifting	1 (1.7)
	Gym	1 (1.7)
	Football	1 (1.7)
	Aerobics	1 (1.7)
	Combined activity	3 (5.1)
	*Smoking habits*	
	*Nonsmokers*	
	Never smoked	20 (34.5)
	Ex-smoker <1 y ago	1 (1.7)
	Ex-smoker >1 y ago	9 (15.5)
	*Smokers*	
	Cigarettes	8 (13.8)
	Hookah	19 (32.8)
	Both	1 (1.7)

Disease history	*Hypertension*	
	≤1 year ago	4 (6.9)
	1-2 years ago	8 (13.8)
	3-4 years ago	9 (15.5)
	≥5 years ago	37 (63.8)
	*Other diseases*	
	Hyperlipidemia	38 (65.5)
	Diabetes	14 (24.1)
	Thyroid disease	9 (15.5)

Antihypertensive medication	*Antihypertensive therapy*	
	No therapy	2 (3.44)
	Mono-therapy	19 (32.75)
	*Combination therapy*	
	2 drugs	22 (37.93)
	≥3 drugs	15 (25.86)
	*Class of antihypertensive drugs*	
	Beta blocker	26 (44.8)
	Angiotensin converting enzyme inhibitor	14 (24.1)
	Angiotensin II receptor blocker	24 (41.4)
	Calcium channel blocker	21 (36.2)
	Diuretics	23 (39.7)
	*Tau/dosing frequency*	
	12 hours/twice daily	10 (17.2)
	24 hours/once daily	42 (72.4)
	When needed	4 (6.9)

**Table 2:** Anthropometric measurements during and after Ramadan.

	Male			Female		
	During Ramadan	After Ramadan	*p* value	During Ramadan	After Ramadan	*p* value
Height (cm)	174.21 ± 1.62			157.43 ± 1.32		
Body weight (kg)	100.39 ± 3.88	101.32 ± 3.96	0.045	82.26 ± 2.23	81.95 ± 2.28	0.247
BMI (kg/m^2^)	32.54 ± 1.09	32.91 ± 1.14	0.037	33.26 ± 0.93	33.22 ± 0.96	0.786
Body fat (%)	31.78 ± 1.48	32.08 ± 1.45	0.544	47.69 ± 1.00	47.21 ± 1.13	0.398
Skeletal muscle (%)	30.88 ± 0.76	30.71 ± 0.72	0.575	22.47 ± 0.45	22.75 ± 0.52	0.442
Resting metabolism (kcal)	1961.25 ± 52.19	1972.00 ± 53.22	0.099	1500.28 ± 25.36	1496.96 ± 25.14	0.307
Visceral fat level	16.62 ± 1.12	17.00 ± 1.13	0.09	11.78 ± 0.60	11.53 ± 0.61	0.090
Waist circumference (cm)	113.85 ± 3.75	115.81 ± 2.73	0.384	106.10 ± 1.96	107.62 ± 3.25	0.609
Hip circumference (cm)	113.74 ± 1.93	115.05 ± 1.99	0.023	116.25 ± 1.81	115.26 ± 1.81	0.091
Waist-hip ratio	0.99 ± 0.02	1.00 ± 0.01	0.768	0.91 ± 0.01	0.93 ± 0.02	0.369

Values presented as mean ± SEM.

**Table 3:** Lipid profile and glycemic levels among participants during and after Ramadan.

	During Ramadan	After Ramadan	*p* value	Normal values
Triglyceride (mg/dl)	261.71 ± 46.54	223.54 ± 26.92	0.169	40–160
Total cholesterol (mg/dl)	189.29 ± 7.15	184.21 ± 7.04	0.288	135–220
HDL cholesterol (mg/dl)	39.07 ± 1.42	39.40 ± 1.37	0.569	35–55
LDL cholesterol (mg/dl)	120.22 ± 7.36	114.80 ± 7.05	0.253	Optimal recommended <130
				Diabetics recommended <100
LDL/HDL ratio	3.49 ± 0.31	3.25 ± 0.29	0.110	0–3.6
Total cholesterol/HDL cholesterol ratio	5.33 ± 0.34	5.08 ± 0.32	0.113	0–5
Non-HDL cholesterol (mg/dl)	150.22 ± 7.36	144.82 ± 7.05	0.254	Reference value <160
				For subjects at high risk of CVD <130
				For subjects at very high risk of CVD <100
Blood glucose (mg/dl)	121.24 ± 36.12	114.59 ± 39.20	0.023	70–120

Values presented as mean ± SEM. Normal values adapted from Saint Joseph Medical Center reports.

**Table 4:** Blood pressure variation in fasting and nonfasting conditions.

	During Ramadan	After Ramadan	*p* value
*Systolic blood pressure (mmHg)*			
Morning (baseline)	128.48 ± 2.72	126.90 ± 1.83	0.362
Afternoon	125.16 ± 2.15	124.61 ± 2.21	0.438
Evening	127.72 ± 2.17	123.64 ± 2.09	0.103
Morning (end)	126.36 ± 2.12	126.33 ± 1.71	0.880
*p* value	0.750	0.615	

*Diastolic blood pressure (mmHg)*			
Morning (baseline)	86.36 ± 1.94	87.43 ± 2.20	0.816
Afternoon	82.68 ± 1.64	85.23 ± 2.03	0.264
Evening	80.38 ± 1.51	81.98 ± 1.71	0.213
Morning (end)	84.03 ± 1.33	83.64 ± 1.52	0.810
*p* value	0.076	0.202	

Values presented as mean ± SEM.

**Table 5:** Heart rate variability analysis during and after Ramadan.

	Afternoon			Evening			Morning			*p* value (within day)	
	During Ramadan	After Ramadan	*p* value	During Ramadan	After Ramadan	*p* value	During Ramadan	After Ramadan	*p* value	During Ramadan	After Ramadan
*RR interval-derived parameters*										
Mean RR interval (ms)	749.96 ± 17.96	768.74 ± 14.92	0.092	707.37 ± 18.81 ^*∗*^^#^	754.51 ± 13.44 ^*∗*^	0.009	817.68 ± 15.37	798.14 ± 15.02	0.181	<0.0001	0.008
SDNN (ms)	58.62 ± 5.24	44.44 ± 3.55	0.013	46.23 ± 5.95 ^*∗*^^#^	40.52 ± 3.38 ^*∗*^	0.574	61.32 ± 4.39	53.36 ± 4.49	0.082	0.011	0.006
PNN50 (%)	9.43 ± 2.40	8.14 ± 1.84	0.704	7.02 ± 2.23	6.35 ± 1.72 ^*∗*^	0.817	10.16 ± 2.44	9.87 ± 2.33	0.711	0.274	0.049
RMSSD (ms)	40.14 ± 7.47	31.83 ± 5.01	0.349	32.37 ± 7.68	27.96 ± 3.97	0.805	34.72 ± 5.13	35.56 ± 5.56	0.984	0.394	0.204
SD1 (ms)	28.38 ± 5.28	22.51 ± 3.54	0.348	22.89 ± 5.43	19.77 ± 2.81	0.805	24.55 ± 3.63	25.14 ± 3.93	0.984	0.394	0.204
SD2 (ms)	75.17 ± 5.86	57.02 ± 4.03	0.006	59.30 ± 6.74 ^*∗*^^#^	52.66 ± 4.16 ^*∗*^	0.565	81.32 ± 5.54	69.43 ± 5.41	0.059	0.003	0.005
SD2/SD1	4.68 ± 0.34	3.53 ± 0.23	0.001	4.51 ± 0.32	3.52 ± 0.25	0.009	4.94 ± 0.40	4.03 ± 0.29	0.086	0.518	0.148
Stress index (pts.)	279.13 ± 32.42	350.43 ± 44.66	0.032	445.69 ± 45.62 ^*∗*^^#^	347.31 ± 35.74 ^*∗*^	0.07	231.33 ± 25.99	261.68 ± 30.99	0.429	<0.0001	0.014

*Parameters spectral analysis*										
Rel. power HF band (%)	16.88 ± 2.60	16.64 ± 2.14	0.918	14.47 ± 2.23	15.53 ± 1.83	0.762	14.35 ± 1.86	19.82 ± 2.82	0.05	0.460	0.155
Rel. Power LF band (%)	27.38 ± 1.88	34.73 ± 2.12	0.003	30.03 ± 1.64	35.55 ± 1.97	0.059	26.52 ± 1.61	29.23 ± 2.26	0.290	0.268	0.070
LF/HF ratio	4.87 ± 0.83	3.41 ± 0.32	0.053	4.93 ± 0.59	4.15 ± 0.47	0.218	4.22 ± 0.62	4.54 ± 0.77	0.764	0.651	0.213
Rhythmisation degree	7.10 ± 0.62	6.76 ± 0.61	0.464	6.00 ± 0.76	5.98 ± 0.46	0.165	6.37 ± 0.51	6.50 ± 0.48	0.710	0.260	0.266

HF band (0.150–0.400 Hz); LF band (0.040–0.150 Hz); VLF band (0.003–0.040 Hz); ULF band (0.0001–0.0030 Hz). Values presented as mean ± SEM. ^*∗*^Statistically different compared to morning period of the same day. ^#^Statistically different to afternoon period of the same day.

## Data Availability

The data that support the findings of this study are available from the corresponding author, upon reasonable request.
